# Association Between Body Mass Index and Intracranial Aneurysm Rupture: A Multicenter Retrospective Study

**DOI:** 10.3389/fnagi.2021.716068

**Published:** 2021-08-16

**Authors:** Sifang Chen, Jianyao Mao, Xi Chen, Zhangyu Li, Zhi Zhu, Yukui Li, Zhengye Jiang, Wenpeng Zhao, Zhanxiang Wang, Ping Zhong, Qinghai Huang

**Affiliations:** ^1^Department of Neurosurgery, The First Affiliated Hospital of Xiamen University, Xiamen, China; ^2^Department of Neurosurgery, Heze Municipal Hospital, Heze, China; ^3^Department of Neurosurgery, Xiamen Key Laboratory of Brain Center, The First Affiliated Hospital of Xiamen University, Xiamen, China; ^4^Department of Neuroscience, School of Medicine, Institute of Neurosurgery, Xiamen University, Xiamen, China; ^5^BE and Phase I Clinical Trial Center, The First Affiliated Hospital of Xiamen University, Xiamen, China; ^6^Department of Neurosurgery, Changhai Hospital, Second Military Medical University, Shanghai, China

**Keywords:** body mass index, intracranial aneurysms, stroke, cerebrovascular disease, subarachnoid hemorrhage

## Abstract

**Background and Aims:** It has recently emerged the concept of “obesity paradox,” a term used to describe an inverse association between obesity and clinical outcomes in cardiovascular diseases and stroke. The purpose of this study was to investigate the association between body mass index (BMI) and the risk of intracranial aneurysm rupture.

**Methods:** In this study, we conducted a retrospective analysis of a prospectively maintained database of patients with intracranial aneurysms from 21 medical centers in China. A total of 3,965 patients with 4,632 saccular intracranial aneurysms were enrolled. Patients were separated into unruptured (*n* = 1,977) and ruptured groups (*n* = 1,988). Univariable and multivariable logistic regression analyses were performed to determine the association between BMI and intracranial aneurysm rupture.

**Results:** Compared to the patients with normal BMI (18.5 to < 24.0 kg/m^2^), the odds of intracranial aneurysm rupture were significantly lower in patients with BMI 24.0 to < 28.0 kg/m^2^ (OR = 0.745, 95% CI = 0.638–0.868, *P* = 0.000) and patients with BMI ≥ 28.0 kg/m^2^ (OR = 0.628, 95% CI = 0.443–0.890, *P* = 0.009). Low BMI (<18.0 kg/m^2^) was not associated with intracranial aneurysm rupture (OR = 0.894, 95% CI = 0.483–1.657, *P* = 0.505). For males, both the BMI 24.0 to < 28.0 kg/m^2^ (OR = 0.606, 95% CI = 0.469–0.784, *P* = 0.000) and the BMI ≥ 28.0 kg/m^2^ (OR = 0.384, 95% CI = 0.224–0.658, *P* = 0.001) were associated with a lower rupture risk, whereas the inverse association was not observed in females. Both the BMI 24.0 to < 28.0 kg/m^2^ (OR = 0.722 for aged 50–60y, 95% CI = 0.554–0.938, *P* = 0.015; OR = 0.737 for aged >60y, 95% CI = 0.586–0.928, *P* = 0.009) and the BMI ≥ 28.0 kg/m^2^ (OR = 0.517 for aged 50–60y, 95% CI = 0.281–0.950, *P* = 0.0034; OR = 0.535 for aged >60y, 95% CI = 0.318–0.899, *P* = 0.0018) was associated with a lower rupture risk in patients aged ≥50 years, whereas the association was not significant in patients aged <50 years.

**Conclusions:** Increased BMI is significantly and inversely associated with saccular intracranial aneurysm rupture in males and patients aged ≥50 years.

## Introduction

Subarachnoid hemorrhage (SAH) is a life-threatening subtype of stroke, leading to loss of many years of productive life owing to the high morbidity and mortality (Macdonald and Schweizer, 2017; Maher et al., 2020). The overall incidence of SAH is ~9 per 100,000 person-years, ranging from 4.2 per 100,000 person-years to 22.7 per 100,000 person-years worldwide (de Rooij et al., 2007). The incidence of SAH is age-related and peaks in the 60s, and elderly patients commonly experience poor outcomes following SAH compared with the young population. Particularly, there is an increase in mean age over time with a remarkable rise in the proportion of octogenarian patients and a reduction in patients younger than 50 years in recent years (Feghali et al., 2021). In general, the rupture of an intracranial aneurysm is the underlining cause of SAH, accounting for about 85% of cases (Macdonald and Schweizer, 2017). Currently, there is high agreement among neurosurgeons that older age, female sex, smoking, hypertension, multiple aneurysms, size, and location are among pivotal risk factors for rupture of an intracranial aneurysm (Wermer et al., 2007; Greving et al., 2014; Wang Y. et al., 2021). However, these well-established risk factors only explain a small proportion of the risk of intracranial aneurysm rupture (Andreasen et al., 2013; Kleinloog et al., 2018). Thus, the prediction of the risk of intracranial aneurysm rupture for individual patients is still poorly explored, and therefore the search for new risk factors is urgently needed to continue, especially for aging patients.

Traditionally, obesity has been regarded as a well-established risk factor for cardiovascular diseases and stroke. Nevertheless, a growing body of evidence has suggested that an “obesity paradox”-namely that there is an inverse association between obesity and clinical outcomes-exists for patients with hypertension (Kleinloog et al., 2018) coronary artery diseases (Uretsky et al., 2007; Wang et al., 2015), atrial fibrillation (Sandhu et al., 2016), intracerebral hemorrhage (Persaud et al., 2019), and ischemic stroke (Rodríguez-Castro et al., 2019; Liu et al., 2021). Likewise, this paradoxical phenomenon was also observed in patients with SAH (Rinaldo et al., 2019; Rautalin et al., 2020), suggesting a potential protective effect of increased BMI on the risk of adverse events after presenting intracranial aneurysms. Moreover, several metabolic abnormalities similar to obesity, including hypercholesterolemia and diabetes mellitus, seem to could reduce the risk of intracranial aneurysm rupture (Lindgren et al., 2013; Vlak et al., 2013). Furthermore, recent population-based cohort studies have demonstrated that overweight or obesity was associated with a reduced risk of SAH in the general population (Sandvei et al., 2012; Kroll et al., 2016). In addition, the “obesity paradox” phenomenon seems to be pronounced in the elderly population (Wang and Ren, 2018). Accordingly, overweight and obesity might be associated with a decreased risk of intracranial aneurysm rupture.

However, previous results of the effect of BMI on the incidence of SAH are inconsistent (Feigin et al., 2005; Sandvei et al., 2012; Kroll et al., 2016; Kawate et al., 2017), especially in the Asian population (Feigin et al., 2005; Kawate et al., 2017). Particularly, the control groups of the previous population-based cohort studies were general populations rather than patients with unruptured intracranial aneurysms, and therefore whether higher BMI is associated with a decreased risk of intracranial aneurysm rupture remains largely unknown. To date, data examining the association between BMI and the risk of intracranial aneurysm rupture are sparse. Considering that over one-third of the world's population is now classified as BMI-defined overweight and obesity and the population is aging worldwide (Chooi et al., 2019), there is an urgent need to understand the effect of BMI on the intracranial aneurysm rupture. We hypothesized that increased BMI is inversely associated with intracranial aneurysm rupture. Therefore, the purpose of this study was to explore the association between BMI and the risk of intracranial aneurysm rupture based on a prospectively maintained database.

## Methods

### Study Population

The study population was from the National Research and Development Project of Intracranial Aneurysms, which was led by Naval Medical University Changhai Hospital and performed at 21 tertiary academic medical centers in China. Patients with saccular intracranial aneurysms registered in this project between 2017 and 2020 were included. The diagnosis of intracranial aneurysms was based on the results of CT angiography (CTA), 3-dimensional time-of-flight magnetic resonance angiography (3D-TOF-MRA), or digital subtraction angiography (DSA). Both ruptured and unruptured intracranial aneurysms were included. Exclusion criteria included non-definitive aneurysms on angiography imaging, feeding artery aneurysms to arteriovenous malformations (AVM), traumatic aneurysms, fusiform aneurysms, and patients whose aneurysms were treated before a presentation. The patients with unruptured intracranial aneurysms were diagnosed incidentally in cerebrovascular angiography during evaluations for headache, dizziness, neurologic symptoms, or screening for other diseases. The diagnosis of aneurysmal subarachnoid hemorrhage (aSAH) was made if CT scans and/or lumbar punctures revealed blood in the subarachnoid space. Patients who presented with aSAH were categorized as harboring a ruptured status. According to the ruptured status, the patients were separated into ruptured and unruptured groups. This study was approved by the local institutional review board and considered minimal risk. Patient informed consent was, therefore, waived by the institutional review board (IRB). All methods were carried out following the Declaration of Helsinki.

### Exposure Assessment

We conducted a retrospective analysis of a prospectively maintained database of patients with intracranial aneurysms from 21 medical centers. Initially, an electronic data capture (EDC) system had been designed using the Delphi method before the study. Based on the EDC system, information on patient demographic and clinical characteristics was collected. The demographic characteristics included age, sex, height, weight, and ethnicity. The clinical characteristics included pre-existing comorbidities (e.g., hypertension, diabetes, polycystic kidney diseases, atrial fibrillation, acute coronary syndrome, heart failure, hyperlipidemia, history of ischemic stroke, genetic diseases, and peripheral vascular diseases), information on smoking, and alcohol use, and aneurysmal parameters. Aneurysmal parameters included the number, maximum size, shape irregularity, and location of intracranial aneurysms. The height and weight had been measured using a validated measuring instrument in each center. BMI was calculated as measured weight in kilograms divided by the standing height in meters squared. We divided BMI into four groups according to the guideline for prevention and control of overweight and obesity in Chinese adults: <18.5 kg/m^2^ (BMI-defined underweight), 18.5 to <24.0 kg/m^2^ (BMI-defined normal weight), 24.0 to <28.0 kg/m^2^ (BMI-defined overweight), ≥28.0 kg/m^2^ (BMI-defined obesity) (Chen and Lu, 2004). For MRA or CTA, three-dimensional models of aneurysms and their surrounding vasculature had been generated using corresponding software in the offline workstation in each medical center. Measurements of maximal neck-width, neck to dome length (length from the neck center to the dome of the aneurysm), and aneurysm width (measured perpendicular to the neck to dome length) had been performed using the standard projection of 2-dimensional conventional angiograms in DSA and 3-dimensional reconstructions in MRA or CTA. The maximum measurement of aneurysm width or aneurysm neck-to-dome length was defined as the maximum aneurysm size. The locations of intracranial aneurysms were categorized as anterior circulation artery and posterior circulation artery. The irregular shape was defined as that the aneurysm fundus was bi- or multi-lobular or small bleb(s) or secondary aneurysm(s) were protruding from the aneurysm fundus (Lindgren et al., 2016; Wang Y. et al., 2021). Based on this definition, the shape irregularity was independently assessed by two attending neurointerventional surgeons (both with over 15 years of experience in neurointerventional surgery) who were blinded to this study, and the inter-reader agreement was good due to their extensive experience in this field. Finally, the shape irregularity of intracranial aneurysms was categorized as regular and irregular shape.

### Statistical Analysis

The sample size was roughly estimated using the method of event per variable (EPV), which recommends that the sample size with logistical regression should be at least ten times the number of predictors. Since there were over 4,000 cases in the EDC system, the sample size was sufficient to achieve the statistical power. The Shapiro-Wilk test, stem-leaf plot, and normal P-P plot were used for the test of data distribution of continuous variables. Continuous variables were expressed as the median [interquartile range (IQR)] or mean [standard deviation (S.D.)], as appropriate. The Student's *t*-test was used for variables with parametric distribution and the Mann-Whitney test for variables with the non-parametric distribution. Categorical variables were expressed as numbers (frequencies), and the differences between the two groups were analyzed using χ^2^-test or Fisher's exact test.

To determine the association between BMI and intracranial aneurysm rupture in the study population, univariable and multivariable logistic regression models were performed. Candidate variables with a *P* ≤ 0.10 in univariable analysis were included in the multivariable model. Given the fact that multiple aneurysms are common in patients with intracranial aneurysms, there is probably some kind of random effect of aneurysmal parameters on the result. Hence, a mixed effect logistic regression model was performed in the multivariable analysis. In this model, three aneurysmal parameters including size, location, and shape of intracranial aneurysms were included in the random effect model and other variables were included in the fixed-effect model. Furthermore, to explore age and sex differences in the association of BMI and intracranial aneurysm rupture, subgroup analyses were performed according to age and sex stratifications. All data were handled and analyzed using SPSS statistic 25.0 (SPSS Inc., Chicago, USA) and R programming environment (R Foundation for Statistical Computing, Vienna, Austria). All statistical significance was defined as *P* < 0.05.

## Results

### Comparisons of Demographic and Clinical Characteristics Between Ruptured and Unruptured Groups

We enrolled 3,965 patients with 4,632 intracranial aneurysms who met our inclusion criteria, of whom 1,977 patients (49.86%) were unruptured and 1,988 patients (50.14%) were ruptured. There were 1,446 males (36.46%) and 2,519 females (63.54%). The average age was (59.26 ± 10.81) years in the study population, the majority of which (82.55%) was aged ≥50 years. Comparisons of demographic and clinical characteristics between ruptured and unruptured groups before matching are presented in Table 1. There were significant differences in age, sex, BMI, drinking, smoking, hypertension, heart failure, hyperlipidemia, history of ischemic stroke, multiple aneurysms, size, shape irregularity, and location between the two groups (all *P* < 0.05).

**Table 1:** Baseline demographic and clinical characteristics between the two groups.

**Characteristics**	**Unruptured (*n_**1**_*= 1,977, *n_**2**_*= 2,270)**	**Ruptured (*n_**1**_*= 1,988, *n_**2**_*= 2,362)**	***P***
Age (years)	59.05 ± 11.14	59.47 ± 10.47	0.225
<50 [*n* (%)]	384 (19.42)	308 (15.49)	0.002
50–60 [*n* (%)]	683 (34.55)	684 (34.41)	
>60 [*n* (%)]	910 (46.03)	996 (50.10)	
Sex [*n* (%)]			
Male	775 (39.20)	671 (33.75)	0.000
Female	1,202 (60.80)	1,317 (66.25)	
Ethnic [*n* (%)]			
Han	1,944 (98.33)	1,939 (97.54)	0.078
Others	33 (1.67)	49 (2.46)	
BMI (kg/m^2^)	24.19 ± 2.60	23.65 ± 2.74	0.000
18.5–23.9 [*n* (%)]	918 (46.43)	1,111 (55.88)	0.000
<18.5 [*n* (%)]	24 (1.21)	29 (1.46)	
24.0–27.9 [*n* (%)]	908 (45.94)	771 (38.78)	
≥28.0 [*n* (%)]	127 (6.42)	77 (3.88)	
Smoking [*n* (%)]	243 (12.29)	320 (16.10)	0.001
Former	32 (1.62)	32 (1.61)	0.001
Current	211 (10.67)	288 (14.49)	
Drinking [*n* (%)]	157 (7.94)	195 (9.81)	0.039
Hypertension [*n* (%)]	883 (44.66)	970 (48.79)	0.009
Diabetes [*n* (%)]	193 (9.76)	90 (4.53)	0.000
Polycystic kidney diseases [*n* (%)]	3 (0.15)	4 (0.20)	1.000
Atrial fibrillation [*n* (%)]	3 (0.15)	2 (0.10)	0.686
Acute coronary syndrome [*n* (%)]	20 (1.01)	28 (1.41)	0.253
Heart failure [*n* (%)]	37 (1.87)	11 (0.55)	0.000
Hyperlipidemia [*n* (%)]	96 (4.86)	14 (0.70)	0.000
Ischemic stroke [*n* (%)]	233 (11.63)	77 (3.87)	0.000
Genetic diseases [*n* (%)]	9 (0.46)	5 (0.25)	0.280
Peripheral vascular diseases [*n* (%)]	12 (0.61)	5 (0.25)	0.087
No. of aneurysms per patient [mean (SD)]	1.15 (0.45)	1.19 (0.52)	0.000
Location of aneurysms [*n* (%)]			
Anterior circulation artery	2,104 (88.72)	1,819 (77.01)	0.000
Posterior circulation artery	256 (11.28)	543 (22.99)	
Size of aneurysm (mm)	4.00 [3.00–5.80]	4.60 [3.40–6.00]	0.000
<5 mm [*n* (%)]	1,477 (65.07)	1,271 (53.81)	0.000
≥5 mm [*n* (%)]	793 (34.93)	1,091 (46.19)	
Multiple aneurysms [*n* (%)]	239 (12.09)	289 (14.53)	0.008
Irregular shape [*n* (%)]	57 (2.51)	414 (17.53)	0.000

*n_1_ represents the number of patients, n_2_ represents the number of aneurysms; BMI, body mass index*.

### Univariable and Multivariable Analyses for the Risk Factors of Intracranial Aneurysm Rupture

The results of the univariable and multivariable analyses for the risk factors of intracranial aneurysm rupture are shown in Table 2. A total of 16 variables (3 variables in the random effect model and 13 variables in the fixed-effect model) were included in the mixed-effect logistic regression model. In the multivariable analysis, older age (OR = 1.448 for >60 years 95% CI = 1.174–1.784), female sex (OR = 1.381, 95% CI = 1.163–1.639), smoking (OR = 1.542, 95% CI = 1.177–2.202), hypertension (OR = 1.392, 95% CI = 1.195–1.623), aneurysm located in posterior circulation artery (OR = 2.293, 95% CI = 1.883–2.793), irregular shape (OR = 7.956, 95% CI = 5.778–10.913, *P* = 0.000), and aneurysm size with ≥5.0 mm (OR = 1.249, 95% CI = 1.077–1.449) were significantly associated with a higher risk of intracranial aneurysm rupture. In contrast, pre-existing diabetes (OR = 0.501, 95% CI = 0.369–0.679), heart failure (OR = 0.404, 95% CI = 0.181–0.899), hyperlipidemia (OR = 0.146, 95% CI = 0.076–0.281), and ischemic stroke (OR = 0.258, 95% CI = 0.188- 0.354) were significantly associated with a lower risk of intracranial aneurysm rupture.

**Table 2:** Univariable and multivariable analyses for the risk factors of intracranial aneurysm rupture.

**Variables**	**Univariable analysis**		**Multivariable analysis**	
	**OR [95% CI]**	***P***	**OR [95% CI]**	***P***
Age (years)				
<50	Reference		Reference	
50–60	1.249 [1.039–1.500]	0.018	1.212 [0.977–1.504]	0.080
>60	1.365 [1.146–1.625]	0.000	1.448 [1.174–1.784]	0.001
Sex				
Male	Reference		Reference	
Female	1.265 [1.112–1.441]	0.000	1.381 [1.163–1.639]	0.000
Ethnic				
Han	Reference			
Others	1.489 [0.953–2.325]	0.080	1.290 [0.780–2.190]	0.153
BMI (kg/m^2^)				
18.5–23.9	Reference		Reference	
<18.5	0.998 [0.577–1.727]	0.996	0.894 [0.483–1.657]	0.505
24.0–27.9	0.702 [0.616–0.799]	0.000	0.745 [0.638–0.868]	0.000
≥28.0	0.501 [0.373–0.674]	0.000	0.628 [0.443–0.890]	0.009
Smoking	1.369 [1.144–1.639]	0.001	1.542 [1.177–2.020]	0.002
Drinking	1.261 [1.012–1.571]	0.039	1.361 [0.978–1.893]	0.068
Hypertension	1.181 [1.042–1.338]	0.009	1.392 [1.195–1.623]	0.000
Diabetes	0.438 [0.339–0.568]	0.000	0.501 [0.369–0.679]	0.000
Heart failure	0.292 [0.148–0.574]	0.000	0.404 [0.181–0.899]	0.026
Hyperlipidemia	0.139 [0.079–0.244]	0.000	0.146 [0.076–0.281]	0.000
Ischemic stroke	0.302 [0.231–0.393]	0.000	0.258 [0.188–0.354]	0.000
Peripheral vascular diseases	0.413 [0.145–1.174]	0.097	0.838 [0.229–3.062]	0.789
Location of aneurysms				
Anterior circulation artery	Reference		Reference	
Posterior circulation artery	2.605 [2.191–3.099]	0.000	2.293 [1.883–2.793]	0.000
Size of aneurysm (mm)				
<5	Reference		Reference	
≥5	1.767 [1.557–2.006]	0.000	1.249 [1.077–1.449]	0.003
Multiple aneurysms	1.282 [1.068–1.540]	0.008	1.359 [1.135–1.629]	0.001
Irregular shape	9.576 [6.992–13.116]	0.000	7.956 [5.778–10.913]	0.000

### Evaluation of the Association Between BMI and Intracranial Aneurysm Rupture

**Figure 1:**
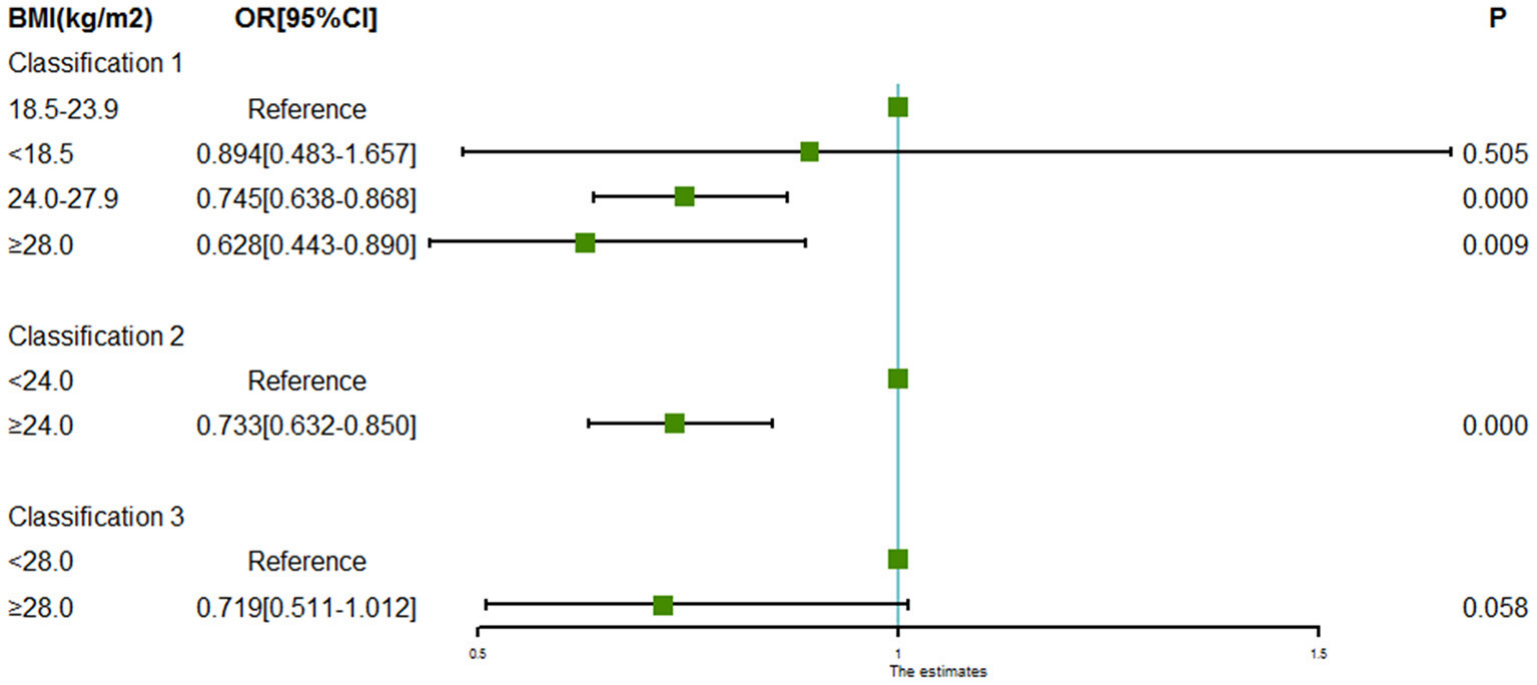
Forest plot of ORs for the association between BMI and intracranial aneurysm rupture. Compared to the patients with normal BMI (18.5 to < 24.0 kg/m^2^), odds of intracranial aneurysm rupture were significantly lower in the patients with BMI 24.0 to < 28.0 kg/m^2^ and patients with BMI ≥ 28.0 kg/m^2^. Low BMI (<18.0 kg/m^2^) was not associated with intracranial aneurysm rupture. Taking the patients with BMI < 24 kg/m^2^ as a reference, the patients with BMI ≥ 24.0 kg/m^2^ had a lower odds of intracranial aneurysm rupture as well.

Evaluation of the association between BMI and intracranial aneurysm rupture is shown in Table 2 and Figure 1. Compared with the patients with normal BMI (18.5 to < 24.0 kg/m^2^), patients with BMI 24.0 to < 28.0 kg/m^2^ (OR = 0.745, 95% CI = 0.638–0.868) and patients with BMI ≥ 28.0 kg/m^2^ (OR = 0.628, 95% CI = 0.443–0.890) had significantly lower odds of intracranial aneurysm rupture. Low BMI (<18.0 kg/m^2^) was not associated with intracranial aneurysm rupture (OR = 0.894, 95% CI = 0.483–1.657, *P* = 0.505). Taking the patients with BMI < 24 kg/m^2^ as a reference, the patients with BMI ≥ 24.0 kg/m^2^ had a lower odds of intracranial aneurysm rupture as well (OR = 0.733, 95% CI = 0.632–0.850, *P* = 0.000).

### Associations Between BMI and Intracranial Aneurysm Rupture Stratified by Sex and Age

**Figure 2:**
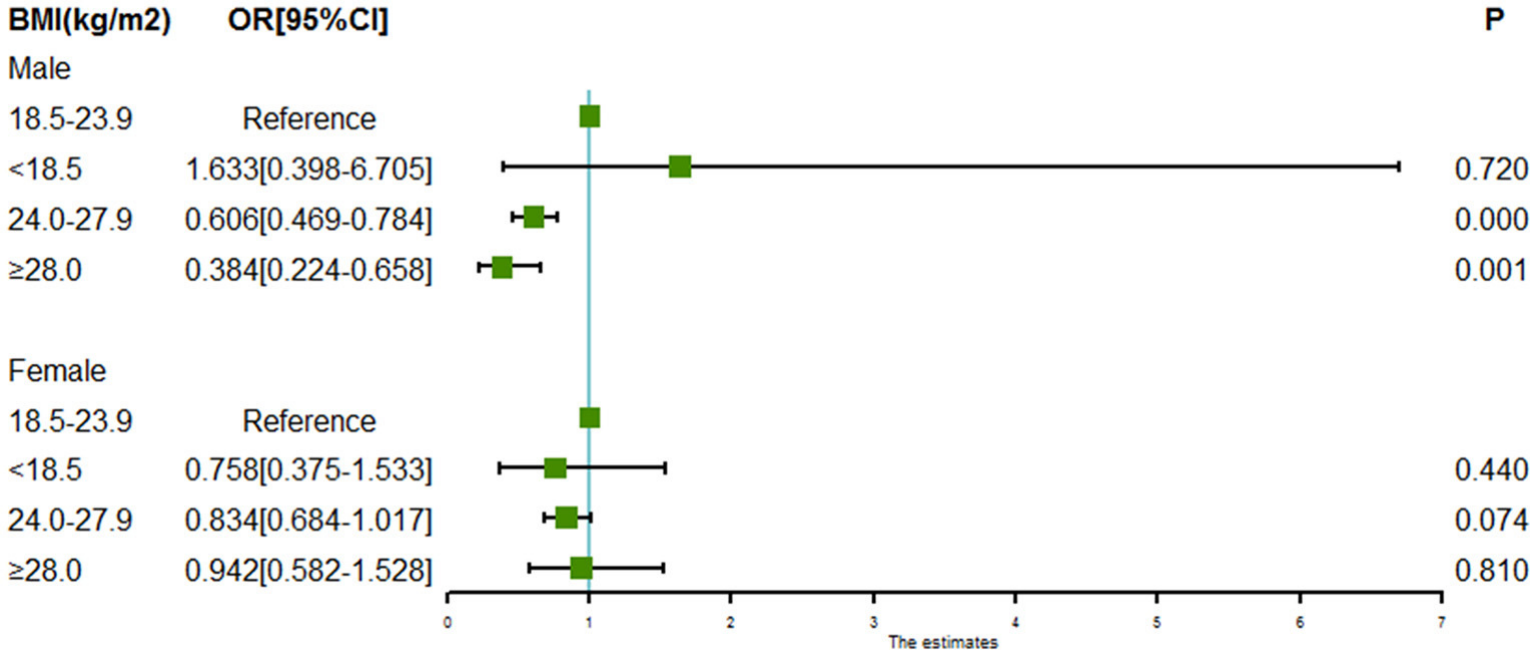
Forest plot of ORs for the association between BMI and intracranial aneurysm rupture stratified by sex. Both the BMI 24.0 to < 28.0 kg/m^2^ and the BMI ≥ 28.0 kg/m^2^ were associated with a lower risk of intracranial aneurysm rupture in males. However, no association was found between BMI and intracranial aneurysm rupture in females.

**Figure 3:**
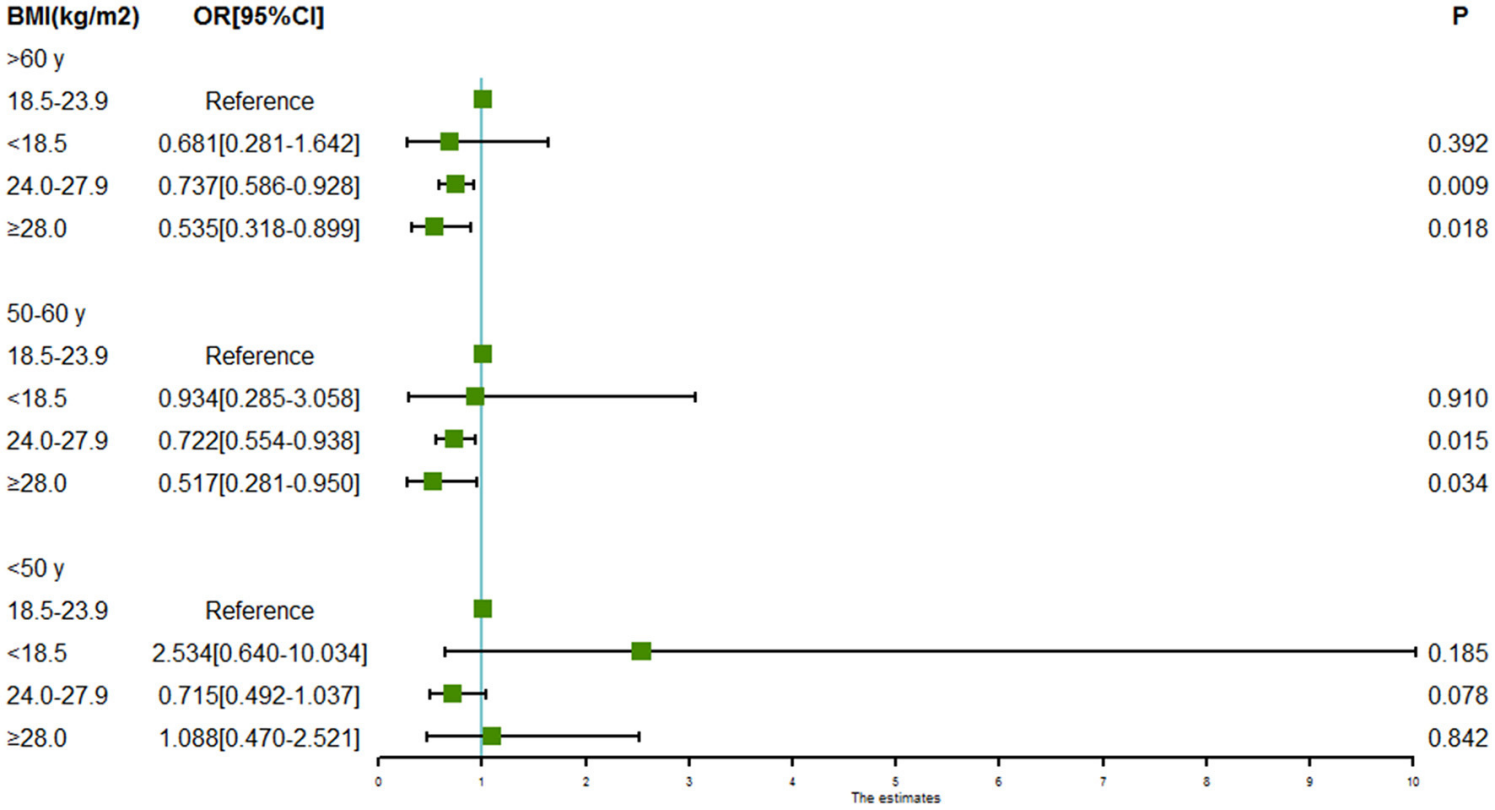
Forest plot of ORs for the association between BMI and intracranial aneurysm rupture stratified by age. Both the BMI 24.0 to < 28.0 kg/m^2^ and the BMI ≥ 28.0 kg/m^2^ were associated with a lower risk of intracranial aneurysm rupture in patients aged 50–60 years and patients aged >60 years. However, no association was found between BMI and intracranial aneurysm rupture in patients aged <50 years.

Subgroup analyses of associations between BMI and intracranial aneurysm rupture stratified by sex and age are shown in Figures 2, 3. For males, both the BMI 24.0 to < 28.0 kg/m^2^ (OR = 0.606, 95% CI = 0.469–0.784, *P* = 0.000) and the BMI ≥ 28.0 kg/m^2^ (OR = 0.384, 95% CI = 0.224–0.658, *P* = 0.000) were associated with a lower risk of intracranial aneurysm rupture. However, no association was found between BMI and intracranial aneurysm rupture in females. Both the BMI 24.0 to < 28.0 kg/m^2^ (OR = 0.722 for aged 50–60y, 95% CI = 0.554–0.938, *P* = 0.015; OR = 0.737 for aged >60y, 95% CI = 0.586–0.928, *P* = 0.009) and the BMI ≥ 28.0 kg/m^2^ (OR = 0.517 for aged 50–60y, 95% CI = 0.281–0.950, *P* = 0.0034; OR = 0.535 for aged >60y, 95% CI = 0.318–0.899, *P* = 0.0018) was associated with a lower rupture risk in patients aged ≥50 years, whereas no significant association between BMI and intracranial aneurysm rupture was found in patients aged <50 years.

## Discussion

In the present study, our results demonstrated that increased BMI was significantly and inversely associated with saccular intracranial aneurysm rupture. Patients with BMI 24.0 to <28.0 kg/m^2^ and those with BMI ≥ 28.0 kg/m^2^ had significantly lower odds of intracranial aneurysm rupture compared with patients with normal BMI (18.5 to < 24.0 kg/m^2^). In particular, this inverse association was significant in males and patients aged ≥50 years, whereas the association was not significant in females and patients aged <50 years. To the best of our knowledge, this is the largest multicenter study to date to investigate the association between BMI and intracranial aneurysm rupture.

In line with existing literature (Wermer et al., 2007; Greving et al., 2014; Lindgren et al., 2016; Wang Y. et al., 2021), our study demonstrated that older age, female sex, smoking, hypertension, large size, shape irregularity, and aneurysms in the posterior circulation artery were significantly associated with a higher risk of intracranial aneurysm rupture. Moreover, since risk factors for rupture of an unruptured aneurysm are theoretically similar to those for aneurysm formation and SAH (Macdonald and Schweizer, 2017), our study importantly extends previous studies of the association between BMI and the incidence of SAH. Consistent with previous studies (Sandvei et al., 2012; Kroll et al., 2016), our result showed that the higher BMI was negatively associated with intracranial aneurysm rupture. In Sandvei et al.'s (2012) study, there was no association between obesity and the risk of aSAH. However, patients with BMI-defined obesity (≥28.0 kg/m^2^) had lower odds of intracranial aneurysm rupture in this study. Particularly, the higher BMI showed a lower OR for the rupture risk, suggesting that this association tends to be linear. In accordance with the present results, Kroll et al. (2016) demonstrated that higher BMI was linearly associated with a decreased risk of SAH. Similarly, one standard deviation higher BMI was reported to be associated with a risk ratio (RR) of 0.94 (95% CI = 0.91–0.99) for the incidence of SAH in a Sweden cohort of 950,000 adults (Sundström et al., 2019).

It is difficult to explain this inverse association. However, there are several possible explanations for this result. First, since BMI does not accurately account for the regional fat distribution, high BMI is not necessarily about being true overweight or obese. Besides, about 12% of obese individuals belong to the metabolically healthy obese individuals (obese but fit individuals) (van Vliet-Ostaptchouk et al., 2014), which have lower cardiovascular diseases risk compared to normal weight but unfit individuals (*the “fat but fit” hypothesis*) (Ortega et al., 2016; Antonopoulos and Tousoulis, 2017). This is the reason that we interpret the inverse association restricting in higher BMI instead of overweight and obesity. Second, the vascular wall mechanical properties, such as wall stress on the lumen, are currently considered to play a key role in the initiation, growth, and rupture of intracranial aneurysms (Turjman et al., 2014). High wall shear stress could promote the migration of smooth muscle cells (SMCs) and phenotypic changes, resulting in smooth muscle cells' secretion of inflammatory mediators and factors involved in the degradation of the vessel wall of the intracranial aneurysm (Staarmann et al., 2019). This process was suggested to be associated with the growth and rupture of the small or secondary bleb aneurysm phenotype of intracranial aneurysms (Meng et al., 2014). Coincidentally, low BMI was reported to be inversely associated with the peak wall stress in patients with abdominal aortic aneurysm (AAA), leading to an increased rupture risk of abdominal aortic aneurysm (Sweeting et al., 2012; Lindquist Liljeqvist et al., 2017). Given this result and the fact that the majority of the study population presented a small aneurysm (size <10 mm), patients with a relatively lower level of BMI might have higher wall shear stress and then increase the rupture risk of small intracranial aneurysms. Third, patients with higher BMI are more likely to take statins for secondary prevention to reduce the risk of cardiovascular diseases and ischemic stroke in clinical practice. The experimental animal models, as well as clinical studies, suggest that statins have various pleiotropic effects including anti-inflammatory and anti-thrombotic that could reduce the risk of intracranial aneurysm rupture (Sweeting et al., 2009; Can et al., 2018). However, the pathophysiological basis accounting for the inverse association remains unclear, and therefore further research is recommended to be undertaken to draw these inferences.

In the subgroup analysis, we found that there was a significant sex difference in the inverse association between BMI and intracranial aneurysm rupture. Our sex-strata result is similar to several previous cardiovascular and cerebrovascular studies where they also observed that the inverse association between BMI and adverse events is more prominent in males than in females (Hong et al., 2018; Liu et al., 2021). This result may be partly explained by the fact that there is a sex difference in the prevalence of obesity. A recent study, based on the China Chronic Disease and Risk Factors Surveillance program, demonstrated that males have significantly higher proportions of overweight and obesity than females in China, especially in recent years (Wang L. et al., 2021). Similarly, male patients had higher proportions of BMI-defined overweight and obesity in the present study (data not showed). Looking back, it makes sense that overweight and obesity were not or even positively associated with the incidence of SAH in a Japanese cohort study due to its disproportionally high percentage of females (Kawate et al., 2017). Besides, it is easy to understand the phenomenon that male sex is related to a lower risk of intracranial aneurysm rupture from the perspective of sex difference in this inverse association. At the same time, our results contrast with the Sweden cohort study where the inverse association of BMI with risk of SAH was found in females (Sundström et al., 2019). In addition to the difference in controls, this inconsistency might be attributed to the regional difference in the epidemiology of obesity as well as the racial difference in the fat distribution between the Asian and Caucasian populations (Deurenberg et al., 2002; Lim et al., 2011). For instance, visceral adiposity, a fat that is thought to be more dangerous than subcutaneous adiposity, was reported to be more prominent in Asian females than Caucasian females with similar BMI (Lim et al., 2011).

Consistent with the literature, the majority of patients with intracranial aneurysms were aged ≥50 years and about half of them were elderly. Interestingly, our results demonstrated that BMI was negatively associated with intracranial aneurysm rupture both in patients aged 50–60 years and those aged >60 years, whereas the inverse association was not found in younger patients. This study supports evidence from previous observations that older individuals have a more significant inverse association between BMI and the incidence of SAH (Sundström et al., 2019). Besides, this result further confirms the phenomenon that the “obesity paradox” seems to be profound in aging patients (Wang and Ren, 2018). Since there is a complex relationship between aging, metabolism, and relevant disease, it is difficult to explain this result. However, one possible explanation for this might be the loss of skeletal mass due to the aging process in older individuals. On the one hand, weight loss, especially for the loss of skeletal mass, is common in the elderly population, and advanced age (>65 years) is particularly vulnerable to loss of skeletal mass (Bischof and Park, 2015), which in turn has been shown to be highly related to the growth of intracranial aneurysms (aneurysmal growth easily leads to a rupture) (Giordan et al., 2018). On the other hand, loss of skeletal mass is thought to be negatively associated with BMI (Iannuzzi-Sucich et al., 2002). In this perspective, elderly patients with relatively high BMI would have less likelihood of loss of skeletal mass, resulting in a lower risk of intracranial aneurysm rupture. Similarly, Kuo et al. (2006) suggested that elderly individuals with elevated BMI present better cognitive performance in terms of reasoning and visuospatial speed of processing than those with normal BMI, in which a high level of skeletal mass was inferred to be an important contributor. Consequently, if loss of skeletal mass is the accountable factor, more efforts to reduce the risk of intracranial aneurysm rupture as well as improve the structure and function of the aging brain, including adequate protein intake and moderate physical exercise, are warranted in aging patients with intracranial aneurysms. However, the impact of a loss of skeletal mass on the intracranial aneurysm rupture is still needed to be validated, and further prospective studies are therefore suggested to be undertaken in the future.

Strengths of our study include the comprehensive collection of directly measured data from prospective hospital-based samples, the large sample size, the many harmonized exposure variables, and a multicenter design, which could largely reduce the likelihood of random error, selection bias, and measurement bias. With an increasing number of BMI-defined overweight and obesity and increasing mean age in patients with intracranial aneurysms in recent years, this study addresses a clinically relevant question for the clinicians. Our findings could help clinicians to fully understand the effect of BMI on the natural history of unruptured intracranial aneurysms, providing important implications in deciding on the optimal management of unruptured intracranial aneurysms in patients with higher BMI, especially for aging patients.

Despite the intriguing findings of the present study, several important limitations should be taken into account. First, our study design is retrospective, which has less power to estimate the cause-effect. Besides, as patients with ruptured intracranial aneurysms might have weight loss owing to the negative nitrogen balance at presentation, the sudden weight loss might have an impact on the results. Despite that, the majority of the study population was admitted to the hospitals within 1 day, and thereby the impact of weight loss on the results could be thought to be minor. In any case, a large prospective cohort study is needed to confirm our results in the future. Second, since nearly one-fourth of patients with SAH would die before admission to a hospital, the cases in the present study may not be fully representative of all cases of ruptured intracranial aneurysms. There is some kind of prevalence-incidence bias (Neyman bias) in the present study. Third, although we adjusted a set of crucial covariates in multivariable models, there are still potential confounders that could influence our results. Particularly, previous SAH is independently associated with intracranial aneurysm rupture. Since the relevant information was not available in the database, this important covariate was not included in the multivariable model. The absence of the important factor in the multivariable model might have an impact on the results. Further studies, which take these variables into account, will need to be undertaken. Fourth, although the loss of skeletal mass was inferred to contribute to the significant inverse association between BMI and intracranial aneurysm rupture in aging patients, no direct evidence could be presented. Thus, to draw this inference, further prospective studies on the exact benefit of life and behavioral change are therefore suggested to be undertaken in the future. Last but not least, the results of the present study were based on the Chinese population. As there are regional and racial differences in the epidemiology of obesity across countries, replication of the results in other populations is suggested. This is an important issue for future research.

## Conclusions

In summary, the findings of our study demonstrated that increased BMI was significantly and inversely associated with saccular intracranial aneurysm rupture in males and patients aged ≥50 years. With an increasing number of BMI-defined overweight and obesity and increasing mean age in patients with intracranial aneurysms in recent years, our findings could provide important implications in deciding on the optimal management of unruptured intracranial aneurysms in patients with higher BMI, especially for aging patients. However, a large prospective cohort study is needed to confirm our results in the future.

## Data Availability Statement

The raw data supporting the conclusions of this article will be made available by the authors, without undue reservation.

## Ethics Statement

The studies involving human participants were reviewed and approved by the Institutional Review Board of the First Affiliated Hospital of Xiamen University. Written informed consent for participation was not required for this study in accordance with the national legislation and the institutional requirements.

## Author Contributions

SC, XC, ZL, JM, YL, ZZ, ZJ, and WZ collected the data and performed the research. SC and PZ analyzed the data and drafted the manuscript. PZ and QH conceived and designed the research. ZW and QH initiated and organized this study. All authors reviewed and edited the manuscript and approved the final version of the manuscript.

## Conflict of Interest

The authors declare that the research was conducted in the absence of any commercial or financial relationships that could be construed as a potential conflict of interest.

## Publisher's Note

All claims expressed in this article are solely those of the authors and do not necessarily represent those of their affiliated organizations, or those of the publisher, the editors and the reviewers. Any product that may be evaluated in this article, or claim that may be made by its manufacturer, is not guaranteed or endorsed by the publisher.
